# High glucose may decrease the innate immune through TLRs in cornea epithelium

**Published:** 2011-12-24

**Authors:** Hailong Ni, Xiaoran Yan, Zhenyun Lin, Xiuming Jin

**Affiliations:** 1 Eye Center, Affiliated Second Hospital, School of Medicine, Zhejiang University, Hangzhou, China; 2 Department of Ophthalmology, Qingdao Municipal Hospital, Qingdao, China; 3 Department of Gynaecology and Obstetrics, The First People's Hospital of Hangzhou, Hangzhou, China

## Abstract

**Purpose:**

The purpose of this study was to investigate the high potential of glucose in inhibiting the innate immune in cultured human cornea epithelial cells (HCEC) and try to determine whether the role of high glucose on the HCEC relate to toll-like receptor (TLR)2 and TLR4.

**Methods:**

Cells were cultured for 3 days in 5 mmol/l (normal glucose). Then high glucose (25 mmol/l) was added along with normal glucose with daily changes in media for 24 h. The cells were also treated with mannitol as an osmotic control. The cellular abundance of the mRNAs for *TLR2* and *TLR4* was determined by real-time polymerase chain reaction analysis. The proteins of TLR2 and TLR4 were also compared by immunofluorescent staining and western blot. The release of interleukin 6 (IL-6) and IL-8 from cultured HCEC was measured using enzyme-linked immunosorbent assays (ELISA) in the presence and absence of specific blocking antibodies to TLR2 and TLR4.

**Results:**

Incubation of HCEC with high glucose showed that **t**he mRNA expression of *TLR2* and *TLR4* was markedly inhibited. Immunofluorescent staining and western blot analysis confirmed that the protein expression of TLR2 and TLR4 was downregulated in response to high glucose. The result of ELISA also showed that the release of IL-6 and IL-8 can be inhibited by high glucose, but these inhibitions were partly counteracted after pretreatment with anti-TLR2 and/or anti-TLR4 monoclonal antibody. The results also showed that the osmotic control did not affect the expression of TLR2, TLR4, and IL-6, 8.

**Conclusions:**

High glucose may decrease the innate immune through TLRs in cornea epithelium.

## Introduction

With rapid increases in the prevalence of diabetes mellitus (DM) worldwide, ocular complications have become a leading cause of blindness in the world [1]. In addition to abnormalities of the retina (diabetic retinopathy) and the lens (cataract), various types of corneal epithelial disorders are also relatively common in persons with DM [2]. Abnormalities of the cornea include defects in epithelium-basement membrane adhesion and altered epithelial functions such as basal cell degeneration [3], superficial punctate keratitis [4], breakdown of barrier function [5], fragility [6], recurrent erosions, and persistent epithelial defects [7]. Epithelial defect may also result in sight-threatening complications, such as stromal opacification, surface irregularity, and microbial keratitis [8].

The cornea epithelial cells constitute the first line of defense against microbial pathogens, possess the ability to detect their presence [9-11], and play an important role in inflammatory responses by releasing various mediators, such as cytokines and chemokines [12,13]. Recently, Toll-like receptors (TLRs) have proven essentialin triggering the innate immune response by recognizing pathogen-associated molecular patterns (PAMP) and stimulating the activity of host immune cells against several microbial products [14]. TLRs are activated by both endogenous and exogenous agonists of microbial and nonmicrobial origin. TLR activation by their agonists triggers a signaling cascade, leading to cytokine production and initiation of an adaptive immune response [15]. TLR2 and TLR4 bind to components of the Gram-positive and -negative bacteria, respectively [15]. They are expressed in multiple cells and tissues, including in corneas. The interactionsbetween inflammation and diabetes have clear implications for the immune system. Mohammad et al. [16] reported increased TLR2 and TLR4 expression in type 1 diabetic non-obese diabetic (NOD) mice, correlating with increased nuclear factor -kappa-B (NF-κB) activation in response to endotoxin, and increased proinflammatory cytokines. Using TLR2−/−, TLR4−/−knockouts, and NOD mice, Kim et al. [17] demonstrated that TLR2 senses β-cell death and contributes to the instigation of autoimmune diabetes. Devaraj et al. [18] showed increased TLR2 and TLR4 expression, intracellular signaling, and TLR-mediated inflammation in monocytes with significant correlation to HbA1c (A1C) levels in type 1 diabetic patients. Also, Creely et al. [19] showed increased TLR2 expression in the adipose tissue of type 2 diabetic patients with strong correlates to endotoxin levels. Taken together, these observations suggest a potential role for TLR2 and TLR4 in the pathology of diabetes.

However, data examining the mechanism of TLR2 and TLR4 expression and function of cornea in diabetes are unknown. Therefore, this study aimed to test the ability of high glucose, one of the key abnormalities of the diabetic condition, to induce TLRs expression in human corneal epithelium.

## Methods

### Reagents and antibodies

Dulbecco's Modified Eagle Medium (DMEM), F12, fetal bovine serum (FBS), glucose, and phosphate-buffered saline (PBS) were obtained from Invitrogen-Gibco (New York, NY). All media and cytokines used for cell culture were endotoxin minimized. Tissue culture dishes and six-well chamber slides were from BD (New York, NY). Affinity purified, monoclonal, antihuman TLR2 and TLR4 and normal mouse immunoglobulin G (IgG) were from eBioscience (San Diego, CA). The second antibody was cy3 from Beyotime Biotechnology (Beyotime, China).

2-(4-Amidinophenyl)-6-indolecarbamidine dihydrochloride (DAPI dihydrochloride) was used to dye the nuclear (Beyotime Biotechnology). Paired antibodies for human IL-6 and IL-8 enzyme-linked immunosorbent assays (EIA) were from BD. RNeasy Mini kit was from Qiagen (Valencia, CA) was used for RNA extraction. RNA PCR kits were from Promega (Fitchburg, WI), Ethidium bromide, DNA molecular size markers and agarose were from Gene Tech (Shanghai, China). SYBR Green PCR Kit was from Applied Biosystems (Foster City, CA).

### Culture of immortalized human corneal epithelial cells

Simian virus (SV) 40 immortalized human corneal epithelial cells (HCEC) were cultured at 37 °C under 95% humidity and 5% CO_2_ in DMEM/F12 containing10% FBS, 5 μg/ml insulin, 0.1 μg/ml cholera toxin, 5 ng/ml human epidermal growth factor, and 40 μg/ml gentamicin supplemented with 5 mmol/l (normal glucose), 25 mmol/l D-glucose (high glucose), or 5 mmol/l D-glucose plus 20 mmol/l mannitol (high mannitol). The cells were kindly provided by Dr Zan Pan (New York University, New York, NY). At last the supernatants were collected and stored at −80 °C after centrifugation to evaluate the releases of IL-6 and IL-8 and the cells were used to extract the mRNA and protein to evaluate the expression of the TLRs.

### Reversed transcript PCR (RT–PCR)

Total RNA prepared from confluent monolayers of HCEC was used to evaluate the expression of *TLR* mRNA. Qiagen RNeasy Mini kit was used for RNA extraction. Total RNA samples were then reverse transcribed in a ﬁnal volume of 20 μl containing 1 µg RNA, 50 mM Tris-HCl, 75 mM KCl, 3 mM MgCl_2_, 5 mM dithiothreitol, 20 U of RNase Inhibitor, 1µl (1μg) random primer, 0.5mM each dNTP and 200 U RNase H-free reverse transcriptase (Promega). The following primer pair was used for the analysis of glyceraldehyde-3-phosphate dehydrogenase (*GAPDH*) by RT–PCR: Upstream 5′-CGG AGT CAA CGG ATT TGG TCG TAT-3′ and Downstream 5′-AGC CTT CTC CAT GGT TGG TGA AGA C-3′.The annealing temperature of the primers was 60 °C. Each PCR was performed in a 20 µl solution containing 1 µl RT reaction products, 10× PCR buffer (2 µl), 1.8 mM MgCl_2_, 0.1 mM each dNTP, 0.1 µM of upstream primer, 0.1 µM of downstream primer, and 0.5 U Taq DNA polymerase. A negative control (the PCR without a preceding RT step) for each sample was run to assess whether there was residual genomic DNA in the DNase-treated samples.

### Real-time PCR

Real-time PCR was performed in an ABI PRISM 7500 Sequence Detection System Thermal Cycler (Applied Biosystems). Real-time PCR was performed on a volume of 15 μl containing 1.5 μl (50 ng) of cDNA and 13.5 μl of master mix containing 7.5 μl of mix (SYBR Green PCR Master Mix; Applied Biosystems), 0.75 μl of each primer (10 pmol/l), and 4.5 μl of diethyl pyrocarbonate–treated water. The primers are listed in Table 1. The program was set at 50 °C for 2 min and 95 °C for 10 min, followed by 40 cycles of denaturation at 95 °C for 15 s, annealing at 60 °C for 60 s. The melting curve was analyzed by elevating the temperature from 60 °C to 95 °C while monitoring ﬂuorescence. SYBR green ﬂuorescence was monitored after each elongation period. Samples were ampliﬁed with *GAPDH* primers for determination of the initial relative quantity of cDNA in each sample, and then all PCR products were normalized to that amount. Negative controls (without template) were produced for each run.

**Table 1:** Primers for real-time PCR .

**Target gene**	**GenBank**	**Forward sequence(5′–3′)**	**Reverse sequence (5′–3′)**	**Amplicon size (bp)**
*TLR2*	NM_003264.3	TCTCCCATTTCCGTCTTTTT	GGTCTTGGTGTTCATTATCTTC	125
*TLR4*	NR_024168.1	GAAGCTGGTGGCTGTGGA	TGATGTAGAACCCGCAAG	213
*GAPDH*	NG_007073.2	CCCCACACACATGCACTTACC	TTGCCAAGTTGCCTGTCCTT	100

Samples were ampliﬁed in triplicates; averages were calculated and differences in C (t) data were evaluated by Sequence Detection Software V1.3.1 (Applied Biosystems). Data analysis used the comparative Ct method (ΔΔCt method) with the following formula: ΔCt=Ct (Target, TLR)-Ct (Endo, GAPDH). The comparative ΔΔCt calculation involved finding the difference between ΔCt of treated cells and the mean value of the ΔCt from the untreated cells. Fold increase in the expression of specific mRNA in treated cells compared to untreated cells was calculated as 2^-(ΔΔCt)^. Data are expressed as RQ (relative quantity) and differences are shown in the ﬁgures as the expression ratio of the normalized target gene, according to the software results.

### Immunofluorescent staining

The HCEC were seeded onto Lab-Tek tissue culture chamber slides without FBS for 24 h. Then the cells were washed with Hank's Balance Salt Solution (Invitrogen-Gibco). The slides were then fixed in 4% paraformaldehyde for 15 min and wash with 10× tris buffered saline (TBS) 3 times for 5 min each time. Then fixed cells were incubated in blocking buffer of 5% BSA (Proliant, Ankeny, IA) and 0.1% Triton X-100 in PBS for 30 min at room temperature. Cells were then incubated with the following dilutions of primary antibodies for 1 h at room temperature: primary mouse anti human TLR2 and TLR4 monoclonal antibodies (20 μg/ml in 5% BSA-PBS) or with mouse IgG (control). The secondary antibodies, conjugated to Cy3, were diluted 1:200 in 5% BSA-PBS and incubated for 1 h at room temperature. Coverslips were washed three times in PBS for 5 min each time, mounted (Vectashield; Vector Laboratories, Burlingame, CA), and viewed with a fluorescence microscope (Zeiss microscope Imager Z1; Zeiss, Oberkochen, Germany). DNA-intercalating dye DAPI dihydrochloride was used for nuclear labeling. For the negative control, preimmune mouse serum was substituted for the primary antibody.

### Western blot

Cells were lysed with radioimmunoprecipitation (RIPA) buffer (150 mM NaCl, 100 mM Tris-HCl [pH 7.5], 1% deoxycholate, 0.1% SDS, 1% Triton X-100, 50 mM NaF, 100 mM sodium pyrophosphate, 3.5 mM sodium orthovanadate, proteinase inhibitor cocktails, and 0.1 mM phenylmethylsulfonyl fluoride [PMSF]), and protein concentration was determined with the bicinchoninic acid (BCA) assay (Micro BCA; Pierce Biotechnology, Rockford, IL). Equal amounts of protein were mixed with SDS–PAGE protein loading buffer and boiled for 5 min. Proteins were separated by sodium dodecyl sulfate–PAGE in Tris/glycine/SDS buffer (25 mM Tris, 250 mM glycine and 0.1% SDS) and electro-blotted onto nitrocellulose transfer membranes. After blocking with 5% nonfat milk, the membranes were incubated overnight with polyclonal antibodies againstTLR2 and TLR4 (1:400 dilution in 5% nonfat milk) in TBST. GAPDH was used as control. After they were washed three times in PBST, membranes were incubated with secondary HRP-conjugated antimouse IgG for 1 h. After the membranes were again were washed with PBST 3 times, 5 min each. Immune complexes were visualized with an enhanced chemiluminescence reagent (Pierce, Rockford, IL). Results were quantified by capturing the exposed X-ray film image and using area measurements from image analysis software.

### Enzyme-linked immunosorbent assay (ELISA)

The concentration of IL-6 and IL-8, in the cell culture supernatant fluids was determined by ELISA. The assay was performed according to manufacturer’s instructions. Results from two representative experiments are presented as the means ±SEM of triplicate cytokine measurements.

### Statistical analysis

Data are expressed as mean±SEM of triplicates from an experiment that was repeated 3 times with similar results. Statistical significance of differences was determined with the non-parametric Wilcoxon test and Student’s *t* test using the SPSS version 11.5 (SPSS, Chicago, IL). Differences were considered statistically significant at p<0.05.

## Results

### Modulation of *TLR* mRNA expression by high glucose

**Figure 1:**
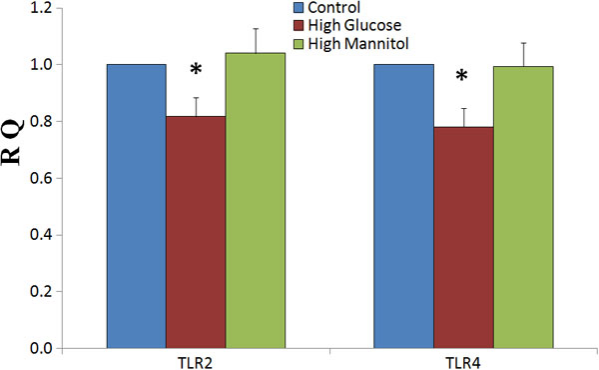
Real time PCR shows the relative expression of *TLR2* and *TLR4* mRNA in high glucose and high mannitol treated HCEC compared with untreated HCEC. The untreated HCEC is regarded as standard control (RQ=1), treated cells are expressed as the multiple of the untreated HCEC. Bars represent means±SEM of 3 independent experiments. *represent a p<0.05 versus control. RQ represent relative quantity.

The results of real-time PCR indicated that high glucose can decrease the mRNA expression of *TLR2* and *TLR4* in cultured HCEC (Figure 1). In contrast, mannitol as an osmotic control had no effect on the mRNA expression of *TLR2* and *TLR4* (Figure 1).

### The expression of TLR2 and TLR4 at the protein level

**Figure 2:**
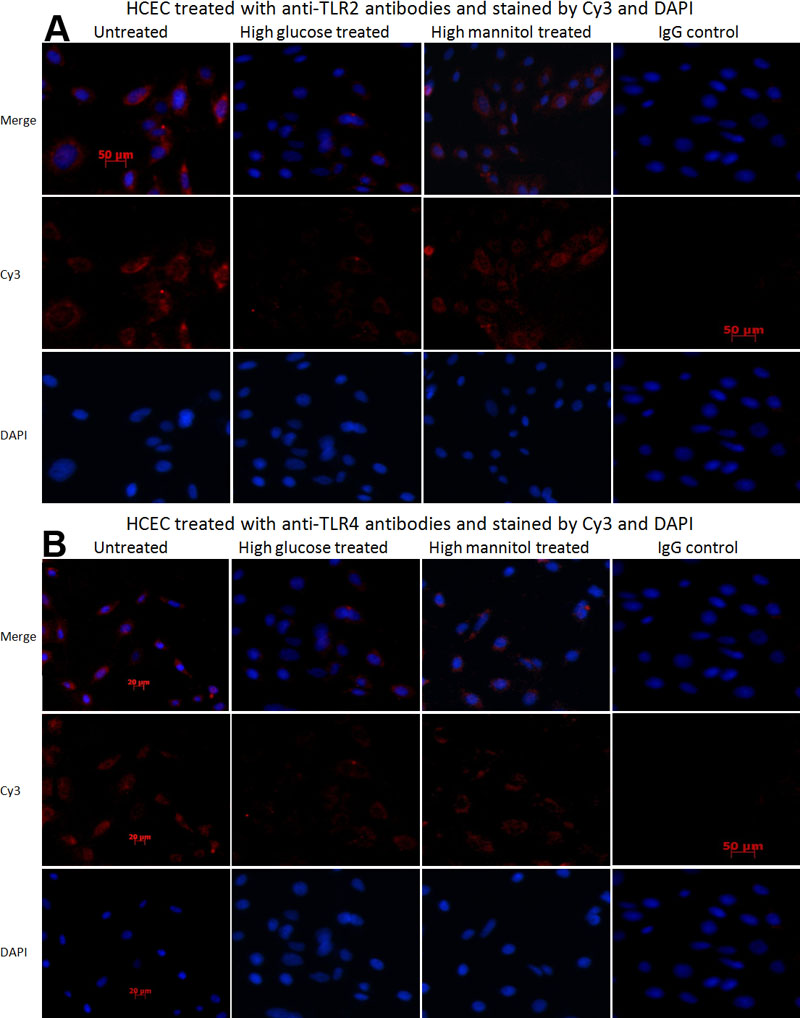
Immunofluorescent staining detect the protein expression of TLR2 and TLR4. The high glucose and high mannitol stimulated cells treated with anti-TLR2 (**A**) and anti-TLR4 (**B**) antibodies and stained by Cy3 and DAPI dihydrochloride. There was no immunoreactivity in the negative control (isotype IgG). Merge means overlap the DAPI and Cy3.

We next evaluated the protein of TLR2 and TLR4 with immunofluorescent staining. Staining was not observed in the cells that were treated with normal mouse IgG (control). Intensity of TLR2 and TLR4 reactivity was slightly inhibited by high glucose, and this inhibition was not observed in mannitol treated HCEC (Figure 2A,B).

**Figure 3:**
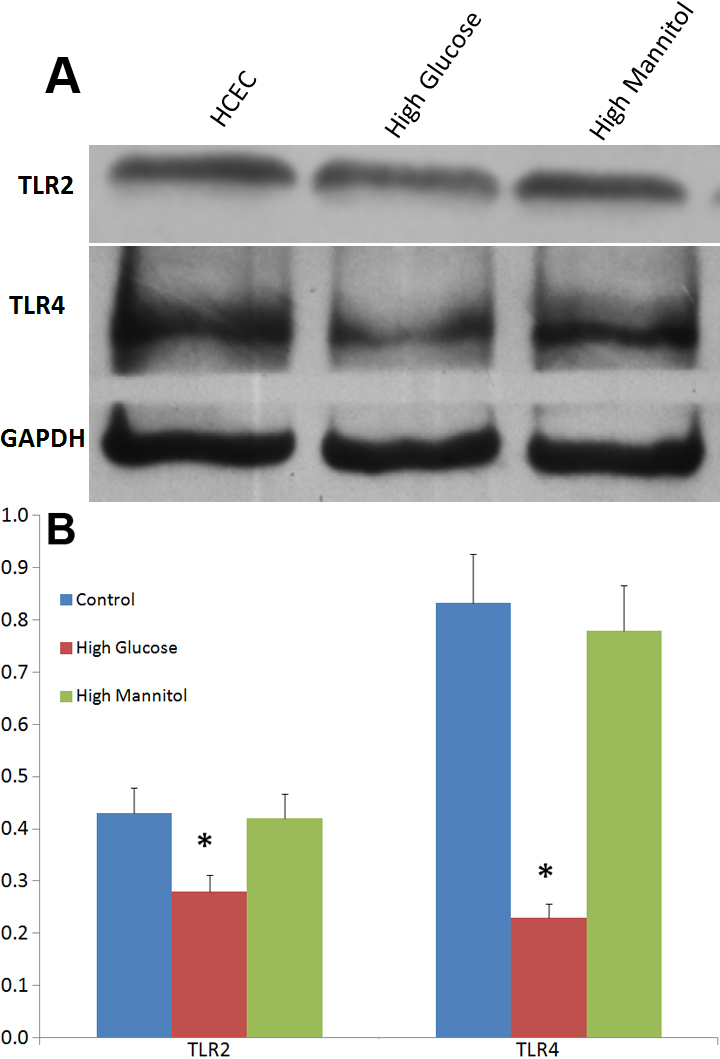
Western blot detect the protein expression of TLR2 and TLR4. **A**: western blot analyses detect the expression of TLR2 and TLR4 at the protein level in HCEC under stimulation of high glucose and high mannitol. Equal amounts of proteins were loaded. **B**: Column diagrams and Bars represent the ratio of the scanned immunoblots of TLR2 and TLR4 to that of GAPDH. Untreated HCEC was regarded as control. Data are the mean±SEM of triplicates from an experiment that was repeated three times with similar results. *represent a p<0.05 versus control.

We also confirmed the protein expression of TLR2 and TLR4 using western blot with a cellular protein GAPDH as standard. The results suggest the protein expression of TLR2 and TLR4 can be inhibited by high glucose, an inhibition was also not observed in mannitol treated HCEC (Figure 3A,B).

### Pretreatment with specific TLR2 and TLR4 monoclonal antibodies counteracted the release of IL-6 and IL-8 inhibited by high glucose

**Figure 4:**
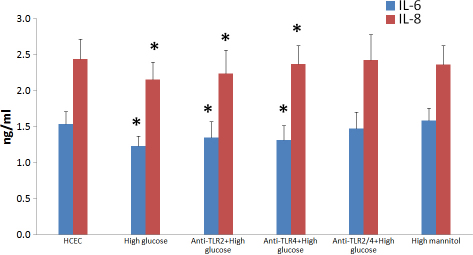
The results of ELISA showed the release of IL-6 and IL-8 from HCEC under high glucose and high mannitol stimulation with and without TLR2 and TLR4 monoclonal antibody (1:200). The untreated HCEC is regarded as control. Data are the mean±SEM of triplicates from an experiment that was repeated three times with similar results. *represent a p<0.05 versus control.

The results of ELISA showed that pretreatment of HCEC with anti-TLR2 and/or anti-TLR4 inhibited the production of IL-6 and IL-8 following exposure to high glucose. Maximal upregulation was observed in HCF cells treated with antibodies against both TLR2 and TLR4. Our results of ELISA also showed that pretreatment of HCEC with anti-TLR2 and/or anti-TLR4 did not inhibit the production of IL-6 and IL-8 exposure to mannitol (Figure 4).

## Discussion

Corneal epithelial cells are constantly exposed to microbial pathogens and their products on the ocular surface. Although the cornea is highly resistant to infections under normal conditions, sight-threatening microbial infections may occur when the corneal integrity is breached by trauma or contact lens wear. Therefore, the underlying mechanisms that regulate corneal epithelial cell activation are important in the development of infectious keratitis. A previous study had showed that high glucose delayed epithelial wound closure in cultured porcine corneas and impaired both basal and wound-induced PI 3-kinase/Akt signaling pathway [20]. Delayed epithelial wound closure may increase risk of the microbial keratitis. As we know, high glucose has been reported to increase cornea opportunity infection by fungi, bacteria, and viruses in clinical observation. However, there is no convincing evidence that high glucose suppresses innate immune responses in the cornea or increase susceptibility to cornea infections. In this study, we investigated the effects of high glucose on the innate immune of corneal epithelium. Our results show that the expression of TLR2 and TLR4 can be inhibited by high glucose. The results suggest that high glucose has the ability to modulate the innate immune response of cornea epithelium.

In this study, we make the novel observation that high-glucose conditions in HCEC inhibit key innate immune system sensors . Our results show that high glucose dose induces a marked decrease in *TLR2* and *TLR4* mRNA and protein expression, and these effects are not osmotic because mannitol had no effect. Signaling through TLRs is believed to be the critical first step in innate immune activation. Increase in TLR2 and TLR4 expression observed here is consistent with those reported in macrophages of atherosclerotic lesions [21], human endothelial cells [22], smooth muscle cells of coronary arteries [23], dendritic cells [24], keratinocytes [25], preadipocytes [26], adipocytes [27], and pancreatic islets [17]. However, all of these studies were performed under nondiabetic conditions with minimal information on the TLR-mediated downstream signaling and mechanism of activation. In this context, we have provided sequential data that in HCEC, high glucose results in decreased TLR2 and TLR4 expression and activity, inhibiting proinflammatory cytokine secretion. Inhibition of TLR2 and TLR4 together in cells was additive, resulting in a further decrease in NF-κB activity and indicating that activation of both receptors is critical.

As we know, recognition of pathogen-associated molecular patterns (PAMP) via TLRs can lead to translocation of the NF-κB, with consequent upregulation of proinflammatory cytokines, co-stimulatory molecules, and chemokines, such as tumor necrosis factor-α (TNF-α), IL-6, IL-8, IL-18, and monocyte chemotactic protein-1 (MCP) [28,29]. Our previous study indicated activation of TLRs in response to *P. aeruginosa* resulted in the upregulation of IL-8, the major chemokine that attracts PMNs into the infected cornea, and of IL-6, which activates polymorphonuclear neutrophils (PMNs) [30]. A similar study also reported poly (I: C) and lipopolysaccharide (LPS) induced IL-6 and IL-8 expression in human corneal epithelial cells [31,32]. Our results show that the expression of TLR2 and TLR4 was inhibited by high glucose. At the same time, the release of IL-6 and IL-8 also showed similar results. The results suggest that high glucose may decrease the host inflammatory response via IL-6 and IL-8. To further prove that the action of high glucose may relate to TLRs, the cultured HCEC was pretreated with specific TLR2 and TLR4 monoclonal antibodies to study the expression of IL-6, 8. Our previous study showed that pretreatment of HCECs with anti-TLR2 or anti-TLR4 inhibited the production of IL-6 and IL-8 following exposure to *Fusarium* hyphae. Maximal inhibition was observed in HCEC treated with antibodies against both TLR2 and TLR4 [33]. That is the reason that both TLR2 and TLR4 were blocked in this study. We found that pretreatment of HCEC with anti-TLR2 and anti-TLR4 upregulated the production of IL-6 and IL-8 which inhibited by high glucose. These results suggest that high glucose may modulate the immune response through TLR2 and TLR4.

Keratopathy in the presence of diabetes should be considered as a potential sight threatening condition and thence must be given appropriate clinical attention and increased research interest. An extensive literature search using MEDLINE found no previous descriptions to study the cornea innate immune of high glucose. Our results suggest that high glucose may decrease the innate immune through TLRs in cornea epithelium. This may be one of the reasons that the risk of cornea infection can be increased in diabetes patients. However, the fundamental question of how high glucose inhibits TLR2 and TLR4 in HCEC and how this leads to decreased inflammation needs further investigation.

One concern has been raised as to why only TLR2 and TLR4 were chosen to study the action of high glucose. TLR2 and TLR4 were chosen based on the following reasons: 1) Among the TLR family, TLR2 and TLR4 has been shown to recognize a wide variety of PAMPs, including bacterial lipoproteins, peptidoglycan (PGN) from bacterial cell wall, and lipoteichoic acids [34-36]. 2) Previous reports on the role of glucocorticoids in innate immunity always focus on TLR2 and/or TLR4 [37-42]. Our results indicated the action of high glucose on cornea innate immune may partly be via TLR2 and TLR4, but whether other TLRs may also play a crucial role needs further investigation.
